# Correlation between plasma ZAG and adiponectin in older adults: gender modification and frailty specificity

**DOI:** 10.1186/s12877-021-02379-4

**Published:** 2021-07-27

**Authors:** Ya-Ping Lee, Chin-Hao Chang, Chin-Ying Chen, Chiung-Jung Wen, Hsien-Liang Huang, Jen-Kuei Peng, Yu-Ting Wang, Ching-Yu Chen, Jaw-Shiun Tsai

**Affiliations:** 1 Division of Family Medicine, Taipei Hospital, Ministry of Health and Welfare, New Taipei City, Taiwan; 2 Department of Family Medicine, National Taiwan University Hospital, 7 Chung-Shan South Road, Taipei, Taiwan; 3 Department of Medical Research, National Taiwan University Hospital, Taipei, Taiwan; 4 Department of Family Medicine, College of Medicine, National Taiwan University, Taipei, Taiwan; 5 Department of Geriatrics and Gerontology, National Taiwan University Hospital, Taipei, Taiwan; 6 Clinical Trial Center, Department of Medical Research, National Taiwan University Hospital, Taipei, Taiwan; 7 Center for Complementary and Integrated Medicine, National Taiwan University Hospital, Taipei, Taiwan

**Keywords:** Adiponectin, Zinc alpha2-glycoprotein, Frailty, Gender differences

## Abstract

**Background:**

Adiponectin and zinc alpha2-glycoprotein (ZAG) are associated with frailty. This study aims to further examine the association of adiponectin with ZAG.

**Methods:**

Outpatients aged 65 years or older with chronic disease followed up in a hospital-based program were recruited for a comprehensive geriatric assessment. We excluded outpatients who were bedridden, residing in a nursing home, with expected life expectancy less than 6 months, or with severe hearing or communication impairment. Plasma ZAG and adiponectin levels were measured. Association between plasma ZAG and adiponectin levels was analyzed by univariate and multivariable linear regression analyses.

**Results:**

A total of 189 older adults were enrolled (91 men and 98 women, mean age: 77.2 ± 6.1 years). Log-transformed plasma ZAG level was 1.82 ± 0.11 μg/mL, and it was significantly higher in men than that in women (1.85 ± 0.12 vs 1.79 ± 0.10 μg/mL, *P* = .0006). Log-transformed plasma adiponectin level was 1.00 ± 0.26 μg/mL, and there was no significant gender difference (*P* = .195). Overall, plasma ZAG level positively correlated with plasma adiponectin level in the multivariable linear regression analysis (*P* = .0085). The gender-specific significance, however, was less clear: this relationship was significant in men (*P* = .0049) but not in women (*P* = .2072). To be more specific by frailty phenotype components, plasma adiponectin was positively correlated with weight loss (*P* = .0454) and weakness (*P* = .0451).

**Conclusions:**

Both of ZAG and adiponectin may be potential frailty biomarkers. Plasma ZAG is an independent factor of plasma adiponectin, especially in older male adults.

## Background

Aging is defined as a series of morphological and functional changes as people age. The change in metabolism is characterized by insulin resistance, changes in body composition, and declines in endocrine functions [1]. Insulin resistance, as a major component of the metabolic syndrome, is a key factor in frailty and sarcopenia and leads to disability, hospitalization, institutionalization, and death [2, 3]. Changes in body composition are mainly characterized by visceral fat accumulation and skeletal muscle loss [1]. Hormones, such as growth hormone, insulin-like growth factor 1, and sex hormones, declines as aging progresses. The changes in hormones in the aging process may cause or contribute to sarcopenia, osteoporosis, and frailty [1].

Adiponectin, mainly derived from adipose tissue, is an important metabolic regulator. Adiponectin has anti-inflammatory, anti-atherosclerotic, and insulin-sensitizing properties [4]. Through the activation of adenosine monophosphate-activated protein kinase (AMPK), adiponectin stimulates glucose uptake and fatty-acid oxidation in skeletal muscle and reduces hepatic gluconeogenesis [5]. Hence, adiponectin is viewed as a “good adipokine” regulating metabolism [6].

Zinc-α2-glycoprotein (ZAG) is initially viewed as a lipid mobilizing factor in cancer cachexia [7]. Recently, ZAG has come to be viewed as a novel adipokine and a potential metabolic regulator associated with adiponectin. Plasma ZAG is low in patients with metabolic syndrome and those newly diagnosed with type 2 diabetes mellitus (DM) [8, 9], and plasma ZAG decreases when the number of metabolic syndrome components increases [8]. In type 2 DM patients, both of plasma ZAG and adiponectin levels increase when glycemic control improves [9]. ZAG expression in adipose tissue is positively correlated with adiponectin expression [10, 11]. Like adiponectin, ZAG is also able to activate AMPK in cultured human skeletal muscle cells [12]. In an experimental study, ZAG treatment reduced blood glucose level and increased expression of the glucose transporter 4 in muscle and adipose tissue of rats [13].

A study conducted to explore the effect of dipeptidyl peptidase-IV (DPP-IV) inhibitor on circulating cytokine levels in newly diagnosed type 2 DM adult patients under 65 years old, found that plasma ZAG is positively correlated with plasma adiponectin [9]. However, the roles of adiponectin and ZAG in metabolism may change as we age. For example, hyperadiponectinemia is associated with poor muscle function and falls in older individuals [14, 15]. Plasma adiponectin and ZAG levels correlate with frailty in older people [16, 17]. Besides, data showed that gender differences may exist. Plasma adiponectin is positively correlated with frailty especially in older male adults [16], and plasma ZAG is positively correlated with frailty especially in older female adults [17]. To understand the relationship between ZAG and adiponectin may be helpful to further explore their role in frailty pathophysiology. Therefore, this study aims to study the relationship between ZAG and adiponectin in older individuals, and to explore the role of gender and frailty on this relationship.

## Methods

### Subjects

From January 2007 to June 2008, outpatients aged 65 years or older with chronic diseases followed up in a hospital-based program were recruited for a comprehensive geriatric assessment if the patients showed functional decline, high healthcare utilization, or otherwise met the inclusion criteria as described previously [16, 17]. Patients who were bedridden, residing at a nursing home, with an expected life expectancy less than 6 months, or with severe hearing or communication impairment were excluded [16, 17].

### Data collection

Experienced research nurses used structured questionnaires to collect basic characteristics including demographics, smoking status, co-morbidity and current medications [16, 17]. Weight was checked with the same machine and blood pressure was measured with a standard sphygmomanometer according to a standard protocol. Two blood pressure data were obtained from the right arm of participants in a sitting position after a 15-min rest at 5-min intervals, and their mean value was calculated. The five frailty phenotype components were assessed by a modified version of Fried’s criteria [16–18] where the definition of “Unintentional weight loss” was modified as more than 3 kg or greater than 5% of the body weight, and “Exhaustion” and “Low physical activity” were modified based on the Depression Scale of Center for Epidemiological Studies [19] and the Taiwan International Physical Activity Questionnaire-Short Form (IPAQ-SF) [20]. The measurements of “Slow walking speed” and “Weakness” were not modified [18]. In measurement of walking speed, the participants were asked to walk 5 m, and the time required to walk that distance was measured to calculate walking speed. Measurement of grip strength was performed by keeping the patient in an upright position with the arms unsupported and parallel to the body. The average of three consecutive measurements obtained at an interval of 30-s rest between each measurement was used for the analysis [16, 17]. The subjects were classified as, “robust” for no positive component, “pre-frail” for 1 or 2 positive components, and “frail” for ≥3 positive components [18].

### Measurement of body composition, biochemical assays, and plasma levels of ZAG and adiponectin

Body mass index (BMI) and waist circumference were measured by experienced research nurses. Appendicular skeletal muscle mass and body fat mass percentage were measured by bioelectric impedance analysis, and appendicular skeletal muscle mass index (ASMI) was calculated as appendicular skeletal muscle mass divided by squared height (kg/m^2^) [17]. In the assessment, the subjects dressed in light clothing, in a fasted state and after voiding, stood on the analyzer barefooted in close contact with the electrodes and grasped both hand holders as shown in the user’s manual.

Blood samples were obtained from the antecubital vein of the subjects after an 8-h fast for complete blood count and biochemical analysis for albumin, total cholesterol, blood urea nitrogen, and creatinine. Plasma ZAG levels and plasma adiponectin levels were measured by commercial enzyme-linked immunosorbent assay (ELISA) kits (BioVendor, Brno, Czech) and a radioimmunoassay method (Linco Research, Inc., St. Charles, MO) [21], respectively. Plasma tumor necrosis factor-alpha (TNF-α) levels were measured by commercial ELISA kits (Assaypro LLC, Saint Charles, Missouri, USA). Plasma C-creative protein (CRP) levels were measured by latex agglutination test (Denka Seiken, Gosen, Niigata, Japan).

### Statistical analyses

Demographic data, body composition, and laboratory tests were summarized as frequencies and percentages for categorical variables and means and standard deviations for continuous variables. T-test and Chi-square test (Fisher’s exact test) were performed to assess the differences between men and women. Linear regression analysis was used to explore the trend between plasma adiponectin levels (log-transformed) and plasma ZAG levels (log-transformed) after adjusted for potential confounders, which were statistically significant (*p* < .05) from the univariate model. A *p*-value < .05 was considered statistically significant. All data were analyzed using SAS 9.4 statistical software (Cary, North Carolina, USA).

## Results

A total of 189 participants were enrolled in this study. Demographic data, body composition, and laboratory tests were summarized in Table 1. There were 91 (48.1%) men and 98 (51.9%) women. The age of all participants was 77.2 ± 6.12 years. Most of the participants never smoked (65.08%), and there was a gender difference in the distribution patterns of smoking status (*P* < .0001).

**Table 1:** Demographic Data and the Results of Physical Examination and Laboratory Tests of the 189 Participants

Variables	Overall^a^ (*n* = 189)	Male^a^ (*n* = 91)	Female^a^ (*n* = 98)	*t(p)* or *χ*^*2*^*(p)*
Age (year)	77.19 ± 6.12	78.05 ± 6.00	76.38 ± 6.15	1.90(.060)
Smoking status				
Never	123 (65.08%)	27 (29.67%)	96 (97.96%)	97.05(<.0001)^b^
Quitted	56 (29.63%)	55 (60.44%)	1 (1.02%)	
Smoking	10 (5.29%)	9 (9.89%)	1 (1.02%)	
Co-morbidity				
Hypertension	159 (84.13%)	73 (80.22%)	86 (87.76%)	2.01(.157)
Hyperlipidemia	115 (60.85%)	50 (54.95%)	65 (66.33%)	2.57(.109)
Diabetes mellitus	79 (41.8%)	36 (39.56%)	43 (43.88%)	0.36(.548)
Coronary artery disease	56 (29.63%)	28 (30.77%)	28 (28.57%)	0.11(.741)
Stroke	51 (26.98%)	26 (28.57%)	25 (25.51%)	0.22(.636)
Medication				
Aspirin	79 (41.8%)	42 (46.15%)	37 (37.76%)	1.37(.242)
β-blockers	45 (23.81%)	22 (24.18%)	23 (23.47%)	0.01(.909)
Calcium channel blockers	89 (47.09%)	41 (45.05%)	48 (48.98%)	0.29(.589)
ACEIs or ARBs	104 (55.03%)	50 (54.95%)	54 (55.1%)	0.001(.983)
Metformin	48 (25.4%)	20 (21.98%)	28 (28.57%)	1.08(.298)
Sulfonylureas	58 (30.69%)	23 (25.27%)	35 (35.71%)	2.42(.120)
Thiazolidinediones	14 (7.41%)	7 (7.69%)	7 (7.14%)	0.02(.885)
Acarbose	6 (3.17%)	3 (3.3%)	3 (3.06%)	0.01(.927)
Repaglinide	5 (2.65%)	2 (2.2%)	3 (3.06%)	0.14(.712)
Statins	65 (34.39%)	24 (26.37%)	41 (41.84%)	5.00(.025)
Frailty Score (Level)^c^				
0 (Robust)	46 (24.34%)	22 (24.18%)	24 (24.49%)	<.0001(.614) ^b^
1 (Pre-frail)	58 (30.69%)	32 (35.16%)	26 (26.53%)	
2 (Pre-frail)	48 (25.4%)	22 (24.18%)	26 (26.53%)	
3 (Frail)	27 (14.29%)	10 (10.99%)	17 (17.35%)	
4 (Frail)	9 (4.76%)	5 (5.49%)	4 (4.08%)	
5 (Frail)	1 (0.53%)	0 (0%)	1 (1.02%)	
Body composition				
BMI (kg/m^2^)	25.05 ± 3.38	24.87 ± 3.52	25.21 ± 3.26	− 0.67(.501)
Waist circumference (cm)	90.29 ± 9.81	90.45 ± 10.04	90.14 ± 9.64	0.21(.833)
ASMI (kg/m^2^)	6.71 ± 1.1	7.53 ± 0.86	5.95 ± 0.66	13.98(<.0001)
Fat mass percentage (%)	34.17 ± 8.15	27.87 ± 5.77	40 ± 5.12	−15.14(<.0001)
Laboratory tests				
Hemoglobin (g/dL)	12.9 ± 1.65	13.34 ± 1.72	12.46 ± 1.47	3.42(.001)
Albumin (g/dL)	4.54 ± 0.34	4.55 ± 0.41	4.54 ± 0.26	0.10(.919)
Total-Cholesterol (mmol/L)	4.87 ± 0.9	4.63 ± 0.79	5.08 ± 0.95	−3.30(.001)
BUN (mmol/L)	7.5 ± 3.4	8.22 ± 4.04	6.83 ± 2.5	2.52(.013)
Creatinine (μmol/L)	113.72 ± 63.51	128.29 ± 57.38	99.5 ± 66.27	2.95(.004)
MDRD-simplify-GFR (mL/min/1.73 m^2^)	57.45 ± 16.94	57.04 ± 17.41	57.85 ± 16.56	− 0.30(.761)
Log (TNF-α (pg/mL))	1.46 ± 0.51	1.45 ± 0.51	1.47 ± 0.5	− 0.30(.762)
Log (CRP (nmol/L))	1.42 ± 0.29	1.44 ± 0.29	1.41 ± 0.3	0.50(.619)
Log (Adiponectin (μg/mL))	1.00 ± 0.26	0.97 ± 0.22	1.03 ± 0.28	− 1.30(.195)
Log (ZAG (μg/ml))	1.82 ± 0.11	1.85 ± 0.12	1.79 ± 0.10	3.51(.0006)

^a^ Data are presented as number (%) for categorical data, mean (SD) for continuous data. ^b^ Fisher’s exact test. ^c^ Scores on a modified version of Fried’s criteria range from 0 (robust) to 5 (frailty)

*ACEIs* angiotensin-converting enzyme inhibitors; *ARBs* angiotensin II receptor blockers; *BMI* body mass index; *ASMI* appendicular skeletal muscle index; *BUN* blood urea nitrogen; *MDRD* modification of diet in renal disease; *GFR* glomerular filtration rate; *TNF-α* tumor-necrosis factor alpha; *CRP* C-creative protein; *ZAG* zinc alpha2-glycoprotein

MDRD-simplify-GFR (mL/min/1.73 m^2^) = 186 × [(CRE)^-1.154^] × [(age)^-0.203^] (if male)

MDRD-simplify-GFR (mL/min/1.73 m^2^) = 186 × [(CRE)^-1.154^] × [(age)^-0.203^] × 0.742 (if female)

The leading co-morbidities of all participants were hypertension (84.13%), hyperlipidemia (60.85%), DM (41.8%), coronary artery disease (29.63%), and stroke (26.08%). The co-morbidities were not significantly different between the male and female subgroups. The leading medicines prescribed of all participants were angiotensin converting enzyme inhibitors (ACEIs) or angiotensin receptor blockers (ARBs) (55.03%), calcium channel blockers (47.09%), aspirin (41.8%), statins (34.39%), and sulfonylureas (30.69%). Most of the medications, except statin (*P* = .025), were not significantly different in male and female. According to the number of frailty phenotype components, 46 (24.34%) were robust, 106 (56.08%) were pre-frail, and 37 (19.58%) were frail. In both of the male and female subgroups, the distribution patterns of frailty severity were similar to that of overall (*P* = .614).

The BMI of all participants was 25.05 ± 3.38 kg/m^2^. There were no differences in BMI or waist circumference between male and female subgroups. ASMI was significantly higher in male than that in female (*P* < .0001), whereas body fat mass percentage was significantly higher in female than that in male (*P* < .0001) (Table 1). Log-transformed plasma TNF-α levels were 1.46 ± 0.51 pg/mL, and log-transformed plasma CRP levels were 1.42 ± 0.29 nmol/mL, similar in men and women (*P* = .762 and .619, respectively). There was no significant difference in log-transformed plasma adiponectin levels between male and female subgroups (0.97 ± 0.22 vs 1.03 ± 0.28 μg/mL, *P* = .195). Log-transformed plasma ZAG levels were significantly higher in male than those in female (1.85 ± 0.12 vs 1.79 ± 0.1 μg/mL, *P* = .0006). Hemoglobin (*P* = .001), total cholesterol (*P* = .001), blood urea nitrogen (BUN) (*P* = .013) and creatinine (*P* = .004) are different between males and females.

**Fig. 1:**
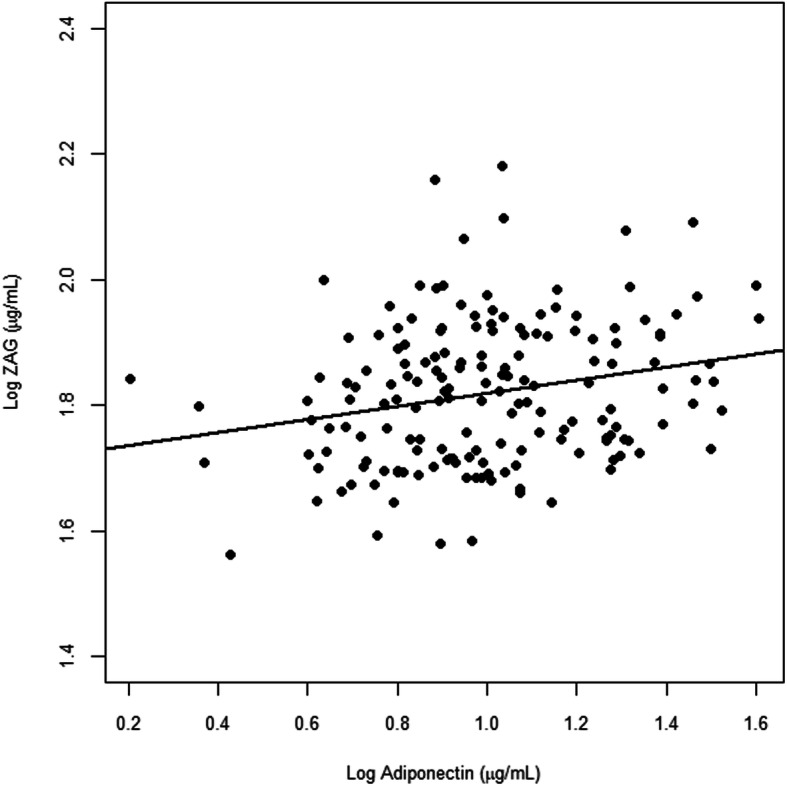
Pearson correlation between log-transformed plasma ZAG and log-transformed plasma adiponectin overall (*p* = .002). *Note*: Log ZAG (ug/ml), log-transformed plasma zinc alpha2-glycoprotein level (ug/ml); Log Adiponectin (ug/ml), log-transformed plasma adiponectin level (ug/mL)

To investigate the overall relationship affecting the levels of plasma adiponectin, we performed univariate and multivariable linear regression analyses on all participants. In the univariate linear regression analysis, log-transformed plasma adiponectin levels were positively correlated with frailty severity, log-transformed plasma ZAG levels, and the use of thiazolidinediones (TZDs), while negatively correlated with BMI, waist circumference, ASMI, the co-morbidities of hypertension and hyperlipidemia (Table 2). However, log-transformed plasma adiponectin levels were independent of fat mass percentage in anthropometric characteristics (Table 2). Further multivariable linear regression analyses on all participants revealed that log-transformed plasma ZAG levels (*P* = .0085), the use of TZDs (*P* = .0044) and the co-morbidity of hypertension (*P* = .0196) were independent factors affecting plasma log-adiponectin levels. Figure 1 showed the correlation between log-transformed plasma ZAG levels and log-transformed plasma adiponectin levels overall (*P* = .002).

**Table 2:** Univariate and Multivariable Linear Regression Analyses for Log-transformed Plasma Adiponectin (μg/mL) in Overall Participants (*n* = 189)

Variable	Univariate			Multivariable		
	Estimate	SE	*P*-value	Estimate	SE	*P*-value
Age (year)	0.00603	0.00323	0.0634			
Sex (female)	0.05127	0.03951	0.1961			
Smoke status	− 0.02789	0.03353	0.4067			
Never	Reference					
Quitted	− 0.04284	0.04372	0.3286			
Smoking	−0.02436	0.08928	0.7853			
Body composition						
BMI (kg/m2)	−0.01790	0.00569	**0.0020**	0.00029	0.00998	0.9765
Waist circumference (cm)	−0.00542	0.00196	**0.0062**	−0.00432	0.00315	0.1728
ASMI (kg/m2)	−0.03647	0.01764	**0.0403**	−0.02178	0.02053	0.2904
Fat mass percentage (%)	−0.00386	0.00244	0.1160			
Co-morbidity						
Hypertension	− 0.11024	0.05183	**0.0349**	− 0.11649	0.04938	**0.0196**
Hyperlipidemia	−0.08334	0.04024	**0.0399**	−0.05997	0.03844	0.1208
Diabetes mellitus	−0.02586	0.04000	0.5188			
Coronary artery disease	−0.07794	0.04229	0.0671			
Stroke	0.05794	0.04460	0.1957			
Medication						
β-blockers	−0.04845	0.04606	0.2944			
Calcium channel blockers	−0.05271	0.03954	0.1843			
ACEIs or ARBs	−0.02609	0.03994	0.5144			
Metformin	−0.05150	0.04497	0.2538			
Thiazolidinediones	0.22701	0.07218	**0.0020**	0.20538	0.07107	**0.0044**
Frail (robust, pre-frail, frail)	0.08571	0.02883	**0.0034**	0.04265	0.02931	0.1476
Log (ZAG (μg/ml))	0.52924	0.17022	**0.0022**	0.45313	0.16997	**0.0085**

*BMI* body mass index; *ASMI* appendicular skeletal muscle index; *ACEIs* angiotensin-converting enzyme inhibitors; *ARBs* angiotensin II receptor blockers

The effects of gender on the plasma adiponectin levels were analyzed (Table 3). In the male subgroup, univariate linear regression analysis showed that log-transformed plasma adiponectin levels were negatively correlated with BMI, waist circumference, body fat mass percentage and the co-morbidity of coronary artery disease, and were positively correlated with frailty severity and log-transformed plasma ZAG levels. In the multivariable linear regression analysis, the co-morbidity of coronary artery disease (*P* = .0029), frailty severity (*P* = .0048), and log-transformed plasma ZAG levels (*P* = .0049) were independent factors affecting log-transformed plasma adiponectin levels. In the female subgroup, univariate linear regression analysis showed that log-transformed plasma adiponectin levels were negatively correlated with body fat mass percentage and the co-morbidity of hypertension while being positively correlated with the use of TZDs and log-transformed plasma ZAG levels. However, there was no significant association between log-transformed plasma adiponectin levels and frailty severity in the female subgroup (*P* = .0673). In the multivariable linear regression analysis, the use of TZDs was the only significant factor, and the relationship between the log-transformed plasma adiponectin levels and log-transformed plasma ZAG levels was not significant (*P* = .2072).

**Table 3:** Univariate and Multivariable Linear Regression Analyses for Log-transformed Plasma Adiponectin (μg/mL) in Males and Females

Variables		Male (*n* = 91)						Female (*n* = 98)				
	Univariate			Multivariable			Univariate			Multivariable		
	Estimate	SE	*P*-value	Estimate	SE	*P*-value	Estimate	SE	*P*-value	Estimate	SE	*P*-value
Age (year)	0.00689	0.00414	0.1001				0.00661	0.00495	0.1857			
Smoke status												
Never	Ref.						Ref.					
Quitted	−0.04086	0.05501	0.4597				0.35729	0.28507	0.2137			
Smoking	−0.05807	0.09122	0.5262				0.32260	0.28507	0.2611			
Body composition												
BMI (kg/m2)	−0.01890	0.00671	**0.0061**	−0.00625	0.01339	0.6419	−0.01703	0.00930	0.0706			
Waist circumference (cm)	−0.00609	0.00233	**0.0105**	0.00195	0.00414	0.6385	−0.00443	0.00318	0.1669			
ASMI (kg/m2)	−0.05127	0.02790	0.0698				−0.04515	0.04755	0.3451			
Fat mass percentage (%)	−0.01311	0.00392	**0.0013**	−0.01063	0.00667	0.1154	−0.01185	0.00590	**0.0478**	−0.00832	0.00571	0.1487
Co-morbidity												
Hypertension	−0.07107	0.06045	0.2432				−0.17674	0.08710	**0.0456**	−0.14848	0.08428	0.0820
Hyperlipidemia	−0.09460	0.04852	0.0546				−0.08333	0.06489	0.2027			
Diabetes mellitus	0.01752	0.04999	0.7269				−0.07227	0.06185	0.2459			
Coronary artery disease	−0.11737	0.05087	**0.0236**	−0.13188	0.04276	**0.0029**	−0.03589	0.06732	0.5953			
Stroke	0.07438	0.05298	0.1642				0.04912	0.07305	0.5032			
Medication												
β-blockers	−0.02968	0.05649	0.6008				−0.06525	0.07290	0.3733			
Calcium channel blockers	−0.07138	0.04874	0.1469				−0.03842	0.06200	0.5372			
ACEIs or ARBs	−0.00162	0.04949	0.9739				−0.05120	0.06242	0.4144			
Metformin	−0.10535	0.05854	0.0756				−0.02100	0.06739	0.7561			
Thiazolidinediones	0.10625	0.09426	0.263				0.32844	0.10712	**0.0029**	0.29821	0.11705	**0.0128**
Frail (robust, pre-frail, frail)	0.08466	0.03657	**0.0231**	0.09375	0.03224	**0.0048**	0.08227	0.04438	0.0673			
Log (ZAG (μg/ml))	0.58753	0.18959	**0.0027**	0.48703	0.16800	**0.0049**	0.69306	0.31878	**0.0326**	0.40493	0.31837	0.2072

*BMI* body mass index; *ASMI* appendicular skeletal muscle index; *ACEIs* angiotensin-converting enzyme inhibitors; *ARBs* angiotensin II receptor blockers

We further analyzed the association of frailty phenotype components with plasma ZAG levels or plasma adiponectin levels (Table 4). We adjusted for age, gender, BMI and DM, and there was no significant association between log-transformed plasma ZAG levels and each frailty phenotype component in overall, male or female adults. Overall, after adjusting for age, gender, BMI and DM, log-transformed plasma adiponectin levels were significantly and positively associated with weight loss and weakness. Furthermore, after adjusting for age, BMI and DM, log-transformed plasma adiponectin levels were only significantly associated with weakness in the male subgroup, while were only significantly associated with slow walking speed in the female subgroup.

**Table 4:** Association between Individual Frailty Components and ZAG or Adiponectin

Variable	All participants			Male (*n* = 91)			Female (*n* = 98)		
	Estimate	SE	*p*-value*	Estimate	SE	*p*-value**	Estimate	SE	*p*-value**
Log (ZAG (μg/ml))									
Weight loss (Yes vs No)	0.02114	0.02754	0.4439	0.00616	0.04443	0.8901	0.04177	0.03251	0.2026
Exhaustion (Yes vs No)	0.02483	0.01813	0.1727	0.01100	0.03074	0.7214	0.03633	0.02017	0.0755
Low physical activity (Yes vs No)	−0.04863	0.03914	0.2158	−0.09031	0.06459	0.1660	−0.00277	0.04535	0.9515
Slow walking speed (Yes vs No)	0.02040	0.01820	0.2638	−0.00151	0.03150	0.9619	0.03918	0.02032	0.0574
Weakness (Yes vs No)	0.01680	0.01791	0.3495	0.02908	0.02901	0.3192	−0.00294	0.02172	0.8927
Log (Adiponectin Levels (μg/mL))									
Weight loss (Yes vs No)	0.12392	0.06147	**0.0454**	0.05998	0.07566	0.4303	0.19057	0.09785	0.0550
Exhaustion (Yes vs No)	0.01041	0.04220	0.8054	0.06142	0.05213	0.2423	−0.03720	0.06595	0.5743
Low physical activity (Yes vs No)	0.09166	0.09086	0.3146	0.18168	0.10989	0.1023	−0.01318	0.14609	0.9283
Slow walking speed (Yes vs No)	0.06625	0.04190	0.1158	−0.01401	0.05383	0.7954	0.13107	0.06479	**0.0464**
Weakness (Yes vs No)	0.08330	0.04126	**0.0451**	0.10670	0.04843	**0.0305**	0.04823	0.07030	0.4947

Note: *ZAG* zinc alpha2-glycoprotein; *BMI* body mass index; *DM* diabetes mellitus

*Adjust for age, gender, BMI, DM

** Adjust for age, BMI, DM

## Discussion

We identified for the first time that plasma ZAG is an important independent factor of plasma adiponectin in the older population, especially in older male adults. This study further finds that, overall, plasma adiponectin positively correlated with weight loss and weakness, and plasma ZAG was not significantly correlated with any of the five frailty phenotype components. Breaking down by gender, plasma adiponectin was positively correlated with weakness in older male adults and with slow walking speed in older female adults, whereas ZAG was not associated with any frailty phenotype component in both older male and female adults. This study demonstrated that the positive relationship between ZAG and adiponectin in the older population and there was a gender difference.

Although the exact role of ZAG in adiponectin regulation remains to be clarified, there is evidence that ZAG might involve in adiponectin regulation and adipose tissue metabolism. In obese individuals, ZAG gene expression in adipose tissue positively correlates with plasma adiponectin [22]. Several in vitro studies in human adipocytes show that recombinant ZAG can enhance adiponectin production [10] and silencing ZAG decreases adiponectin expression [23]. Investigations on the effect of ZAG on adipose tissue give insights into its potential function with a link to adiponectin. Therefore, we speculate that ZAG might involve in adiponectin regulation. Whether ZAG regulates adiponectin via autocrine/paracrine and/or endocrine pathway is needed to be further studied.

Several strands of evidence show that adiponectin and ZAG may involve in changes in muscle strength and muscle function as we age. The present study showed that plasma adiponectin levels were positively correlated with weight loss and weakness in the older Taiwanese individuals. High plasma adiponectin level has been reported to be an indicator of decreased muscle strength of the lower extremity and incident falls in the older Japanese people [14, 15]. The association between adiponectin and changes in muscle strength and muscle function in the older population may be explained by intramyocellular adiponectin and intramyocelluar lipid (IMCL). It was known that different muscles had different proportion of slow-twitch and fast-twitch fibers [24]. Krause MP et al. further found that in mouse skeletal muscles, slow-twitch fibers and fast-twitch fibers consisted of different proportion of intramyocellular adiponectin expression and IMCL content, and this phenomenon may influence muscle function [25]. Thamer C. *et. al*. found that in 63 healthy individuals (age 29.5 ± 0.8 years), plasma adiponectin was negatively correlated with IMCL in soleus muscle but not in tibialis anterior muscle [26]. These data hinted that in different types of muscle, adiponectin has different role in lipid oxidation and may further influence muscle functions. Our data suggested the role of adiponectin in muscle metabolism may change as we age and the mechanism is needed to be investigated.

The effect of ZAG on skeletal muscle has also been investigated. Russel *et. al.* found that *ob/ob* mice treated with ZAG showed a significant increase in the gastrocnemius muscle mass, but not in the soleus muscle [27]. The different effect of ZAG on different muscle may be also due to different muscles had different proportion of slow-twitch and fast-twitch fibers. Besides, one study using needle biopsy from human lateral gastrocnemius muscle showed that the percentage of Type I, Type IIa and Type IIb fibers did not differ with age [28], but muscle capillarization and mitochondrial enzyme activities were significantly lower in older individuals [28]. Furthermore, we have found that in older individuals plasma ZAG positively correlated with ASMI [17], and found that in the present study plasma ZAG positively correlated with slow walking speed in female older adults. These data suggested that the role of ZAG in muscle metabolism may change as we become older and may influence muscle functions. The mechanism how ZAG involves in muscle as we age is also needed to be further studied.

It has been noticed that gender difference in age-associated fat distribution, adipokine secretion and smoking behaviors [29–33]. Our study revealed that, while overall the levels of circulating adiponectin and ZAG were associated, similar patterns of gender differences still existed in the older population. Among the relationship between plasma adiponectin ZAG and frailty phenotype components in this study, gender differences exist. Gender differences in the relationships between adiponectin, ZAG, and frailty have also drawn attention in the literature. That is, the positive relationship between plasma adiponectin and frailty in male older adults [16], and the positive relationship between plasma ZAG and frailty in female older adults [17]. In addition, this study also showed gender difference in body composition and smoking status. In order to exclude the effect of body composition and smoking status on the relationship between plasma adiponectin and ZAG, we use univariate and multivariable linear regression analyses and find that gender difference in the relationship between adiponectin and ZAG still exists after adjusting for body composition and smoking status. Therefore, we speculate that sex hormone might have an important role on the gender difference in the relationship between adiponectin and ZAG.

Previous studies have reported sex hormones might regulate adiponectin and ZAG. For example, testosterone reduces plasma adiponectin levels in men [34]. Testosterone suppresses rat adipocyte to secret the high molecular weight form of adiponectin in the transcriptional process [35]. Also, estrogen and testosterone have the opposite effects on adiponectin synthesis in white adipocytes in vitro [36]*.* Moreover, Cao R. *et. al.* found that ZAG was an androgen-responsive gene and induced cell growth, migration and invasion of prostate cancer cell [37]. How the changes in sex hormone modulate plasma adiponectin and ZAG in the older population requires further investigation.

On the other hand, this study showed no differences between males and females in adiponectin levels. Although several studies reported women had higher adiponectin levels than men, the results akin to ours have been published elsewhere [38]. Possibly for the small sample size in our study, our study showed no differences between males and females in adiponectin levels.

Our study has a few limitations. First, the small sample size limits the number of covariates. A large-scale study is needed to allow for more covariates and to confirm the results obtained here. Second, plasma adiponectin consists of several isoforms that may have slightly different functions. For example, the high-molecular-weight form exerts a protective role as an antidiabetic and anti-atherogenic hormone [39, 40], and the low-molecular-weight form may have a cardiovascular protective role in aging [41]. This study did not distinguish between different adiponectin isoforms. Third, some studies showed that plasma ZAG does not correlate with ZAG expression in visceral adipose tissue [11]. However, we did not investigate the expression of ZAG and adiponectin in adipose tissue in this study. Fourth, previous studies indicated that Caucasian adults have higher circulating adiponectin levels than those in African-American, Hispanic, and Asian [41–44]. Our studies were carried out on an older Asian population, and whether or not there is population variation requires further investigation. We used bioelectric impedance analysis to measure muscle and fat because it was user-friendly and low-cost, although the reliability can be affected by subject, operator, electrodes, and environment [45]. For frailty criteria, we only used Fried’s criteria for the so-called physical frailty and did not explore psychosocial aspects of frailty. Finally, although this study showed positive association between plasma ZAG and plasma adiponectin in older people, it remains unsure whether ZAG and adiponectin indicate the potential for developing frailty in an individual. Further study is needed to clarify the role of ZAG in adiponectin regulation, adipose tissue and muscle metabolism.

## Conclusions

Plasma ZAG is an important independent factor affecting plasma adiponectin in the older population, especially in male older adults. Our findings support the importance of correlation between circulating adiponectin and ZAG in frailty, and may shed some light on the pathogenesis of frailty. The difference between male and female suggests that there are some gender-specific mechanisms for the regulation of circulating adiponectin and ZAG levels. Further studies are necessary to clarify such underlying mechanisms.

## Data Availability

The datasets used or analyzed in the current study are available from the corresponding author on request.

## Abbreviations

AMPK: Adenosine monophosphate-activated protein kinase
ASMI: Appendicular skeletal muscle mass index
BMI: Body mass index
CRP: C-creative protein
DM: Diabetes mellitus
DPP-IV: Dipeptidyl peptidase-IV
ELISA: Enzyme-linked immunosorbent assay
IPAQ-SF: International physical activity questionnaire-short form
TNF-α: Tumor necrosis factor-alpha
TZDs: Thiazolidinediones
ZAG: Zinc-α2-glycoprotein
